# “PERLE bedside-examination-course for candidates in state examination” – Developing a training program for the third part of medical state examination (oral examination with practical skills)

**DOI:** 10.3205/zma001054

**Published:** 2016-08-15

**Authors:** Anne Karthaus, Anita Schmidt

**Affiliations:** 1 Friedrich-Alexander-University Erlangen-Nuremberg, Tutor at the Skills Lab PERLE, Erlangen, Germany; 2 Friedrich-Alexander-University Erlangen-Nuremberg, Head of the Skills Lab PERLE, Erlangen, Germany

**Keywords:** Case-based learning, course in preparation for the state examination, course conception, practical examination

## Abstract

**Introduction: **In preparation for the state examination, many students have open questions and a need for advice. Tutors of the Skills Lab PERLE-„Praxis ERfahren und Lernen“ (experiencing and learning practical skills) have developed a new course concept to provide support and practical assistance for the examinees.

**Objectives:** The course aims to familiarize the students with the exam situation in order to gain more confidence. This enables the students to experience a confrontation with the specific situation of the exam in a protected environment. Furthermore, soft skills are utilized and trained.

**Concept of the course:** The course was inspired by the OSCE-model (Objective Structured Clinical Examination), an example for case-based learning and controlling. Acquired knowledge can be revised and extended through the case studies. Experienced tutors provide assistance in discipline-specific competencies, and help in organizational issues such as dress code and behaviour.

**Evaluation of the course:** An evaluation was conducted by the attending participants after every course. Based on this assessment, the course is constantly being developed. In March, April and October 2015 six courses, with a total of 84 participants, took place. Overall 76 completed questionnaires (91%) were analysed.

**Discussion: **Strengths of the course are a good tutor-participants-ratio with 1:4 (1 Tutor provides guidance for 4 participants), the interactivity of the course, and the high flexibility in responding to the group's needs. Weaknesses are the tight schedule, and the currently not yet performed evaluation before and after the course.

**Conclusion: **In terms of “best practise”, this article shows an example of how to offer low-cost and low-threshold preparation for the state examination.

## Zusammenfassung

**Einführung: **Viele Studierende haben Fragen und Beratungsbedarf bei der Vorbereitung für das letzte mündliche Examen. Um sie bei den Examensvorbereitungen zu begleiten und praktische Hilfestellung zu geben, wurde von Tutorinnen und Tutoren des SkillsLabs Perle- „Praxis ERfahren und LErnen“ der FAU Universität Erlangen-Nürnberg ein neues Kursmodell konzipiert.

**Ziele: **Der Kurs soll den Studierenden helfen, sich mit der Prüfungssituation vertraut zu machen und Sicherheit zu erlangen. So ist die Auseinandersetzung mit dieser spezifischen Situation in einem geschützten Rahmen möglich. Außerdem findet eine Anwendung und eine Vertiefung von Softskills statt.

**Kurskonzept: **Dieser Kurs ist angelehnt an das OSCE Modell (Objective Structured Clinical Examination), einem Fallbasierten Lern- und Prüfungsmodell. Anhand von Fallbeispielen wiederholen und vertiefen die Studierenden bereits gelerntes Wissen. Dabei werden sie von erfahrenen Tutoren begleitet, die Hilfestellung zu fachspezifischen und organisatorischen Fragen wie Kleiderordnung und Verhaltensweisen geben.

**Auswertung des Kurses: **Der Kurs wurde von den Teilnehmern am Ende jedes Kurses evaluiert. Anhand der Evaluation wird der Kurs stetig weiterentwickelt. Im März, April und Oktober 2015 fanden sechs Kurse mit in Summe 84 Teilnehmern statt. 76 Fragebögen (91%) wurden vollständig ausgefüllt und ausgewertet.

**Diskussion: **Als Stärken des Kurses sind ein guter Tutoren-Teilnehmer Schlüssel mit 1:4 (1 Tutor betreut 4 Teilnehmer), die Interaktivität des Kurses und die hohe Flexibilität auf die einzelne Gruppe eingehen zu können, zu nennen. Schwächen sind der limitierte Zeitrahmen und die bisher nicht erhobene Leistungsüberprüfung vor und nach dem Kurs.

**Schlussfolgerung:** Dieser Artikel zeigt im Sinne eines „best practice“-Beispiels eine Möglichkeit auf, niederschwellig und mit geringen Kosten eine Vorbereitung für das praktische Examen anzubieten.

## 1. Introduction

In a newly developed course based on the OSCE model, practical year students are prepared for the last part of the state examination, the oral examination. Tutors of the Skills Lab Perle developed the course “PERLE-Bettenprüfung” (“PERLE bed-side examination”) in order to meet the needs of students just before their third part of the medical state examination. Impetus was the examinees` strong demand for a revision in practical skills and tips for the oral examination. The name of the course is derived from our Skills Lab's name: PERLE – “**P**raxis **ER**fahren und **LE**rnen”, which stands for PERLE – “experiencing and learning practical skills”. The other part of the name was derived from the colloquial word for one part of the last oral examination: “Bettenprüfung” (bed-side examination). In Germany the third and also last state examination is an oral examination, in groups of four students, which takes two days. Four auditors evaluate the student's skills in their own field: Surgery, internal Medicine, one subject chosen by the student and one randomly assigned subject. Besides the theoretical knowledge, practical knowledge is often assessed.

## 2. Objectives

Objectives of the course are training and deepening the use of soft skills, such as talking to patients, giving notice of the following examinations and explaining the further procedure. The course aims to support students through familiarizing them with the exam situation and to gain more confidence. Skills that have been trained during the curriculum are now being performed in a simulated assessment. This enables the students to experience a confrontation with the specific situation of the exam in a protected environment. It was specially developed to provide support to those students who yet feel insecure about the practical part of the third state examination.

## 3. Concept of the course

The structure of the course was inspired by the OSCE model (Objective Structured Clinical Examination). Participants pass through several stations, working on different case studies at each station. The case studies were specially prepared for the course by tutors, taking recent guidelines into account. Medical specialists did the counterchecking. 

Skills in physical examination and anamnesis are assessed. Auditors follow the check-lists to ensure that all the necessary steps are being performed at each station.

In our course we modified the model of OSCE so that student pass through the stations in groups of four. Thirty minutes are planned for each station, so there is time to talk over the case studies and to respond to individual questions.

**Figure 1:**
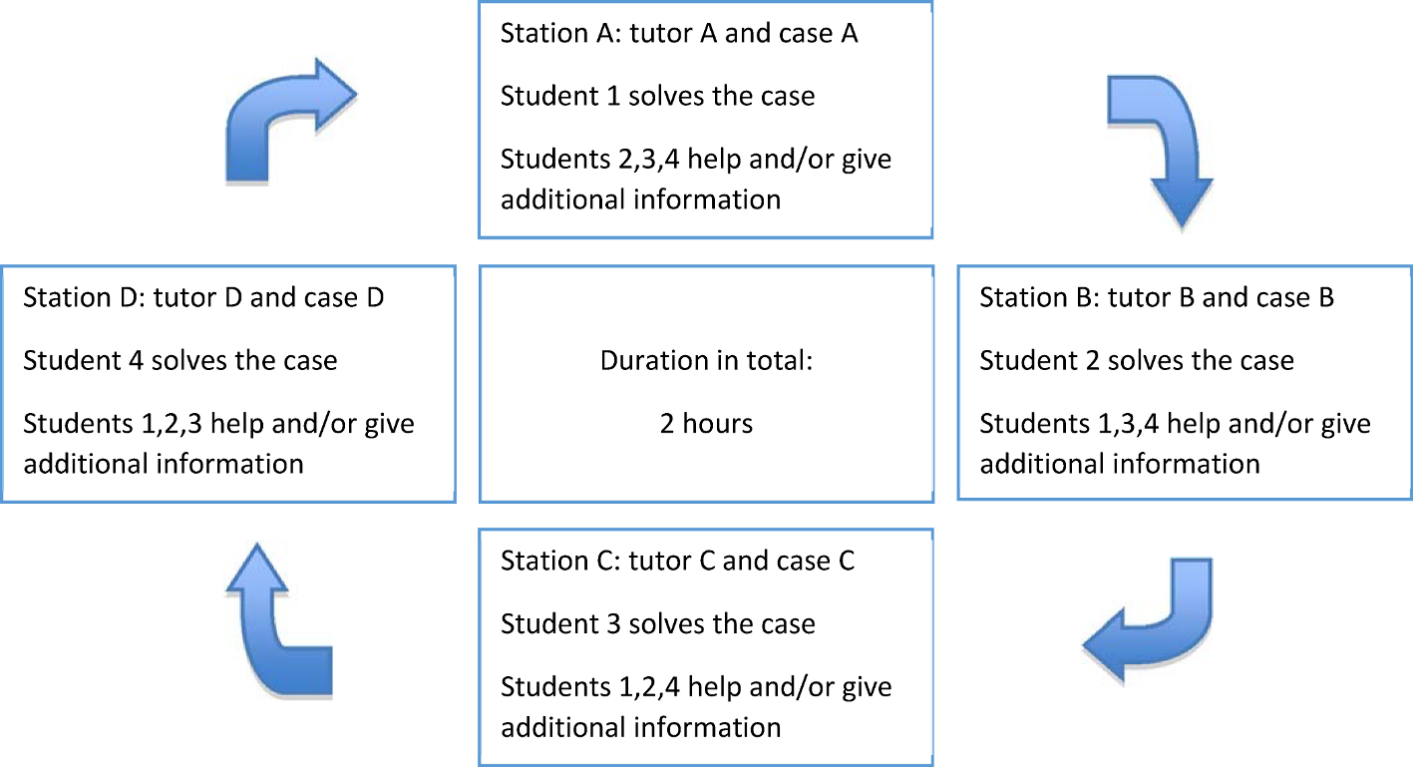
Course structure

Every small group consists of four students (student [1], [2], [3], [4]), which pass through four stations (station A, B, C, D) together. At each station one of the students assumes the role of the doctor and tries to solve the case. The other participants observe the situation and give advice if necessary. Afterwards they give feedback to the “doctor”. In the end every participant is supposed to have worked on one case himself and to have given advice and feedback at the other three stations (see Figure 1 (Fig. 1)). At each station the tutor guides through the case and provides assistance if necessary. The tutor reads out the description of the case report and then starts with the assessment by ticking off the check-list. Afterwards the check-list is discussed (see Attachment 1), and constructive feedback is given to the “doctor”. The different stations cover a variety of medical fields, with a case from neurology, surgery/orthopaedics and internal medicine in each course. Basic components of the physical examination are the same at each and every case-study. At the beginning of the examination the hand disinfection is performed, followed by welcoming the patient and taking the anamnesis.

Furthermore, informative sheets with information on the dress code (see Attachment 2) and a list with “Do´s and Don´ts” (see Attachment 3) are handed out. Tutors of the PERLE have collected information and statements in meetings with professors that have been asked about dress code and behaviour of the examinees. The idea came up, because many students in former courses had questions about some practical advice on these issues. 

## 4. Evaluation of the Course

In the end of every course a written evaluation (see Attachment 4) was performed, in which we asked for the opinion of every student on the following points:

- feedback received from the tutor
- help received from other students
- information given on dress code and “Do´s and Don’ts”
- simulation of the state examination
- assessment of the ease/difficulty and the usefulness of each case study
- wishes and ideas for following courses

The evaluations were given to the participants directly after the course. In numbers, there were six courses, with a total number of 84 students, in March, April and October of 2015.

76 questionnaires were filled in and analysed. 55 were filled in by female students (72,4%) and 21 by male students (27,6%). Item 1 was determined as “too easy/ doesn’t make sens /not helpful” and 5 was determined as “too difficult/ makes sense/helpful”.

The evaluation stated the following: The lists with “Do´s and Don’ts” were evaluated with an average value of 4,55 to be very helpful. Likewise, the information about the dress code was helpful with a mean of 4,1. The simulation of the state examination scored an average value of 4,7, the help received from other students scored 4,4 and feedback received from the tutor 4,8 (see Table 1 (Tab. 1)). 

The case study of acute pancreatitis was evaluated to be very useful with a score of 4,85, although it was rated as easy with 2,95. The case study for hyperthyreosis was said to be very beneficial, with 4,78, and a normal difficulty level of 3,05. Furthermore, the case study of Morbus Bechterew scored 4,4, but was the only one rated a little bit too difficult with 3,5 (see Table 2 (Tab. 2)). 

In the free comment section, the students could remark compliments, critics and wishes for the further development of the course. On the one hand, there has been the wish for more case studies from the field of neurology and orthopaedics, especially the clinical examination of major joints. On the other hand, the students wanted to have more time for the case studies, for the demonstration of the clinical examination by the tutor, and for more questions about theoretical knowledge. Furthermore, they wanted to be able to choose a special field of medicine for their case studies themselves. It was also mentioned that this course should start in earlier years of study with less difficult case studies and then build up to the last course in the practical year. Besides the participants liked the checklists being handed out to them.

Moreover, they liked the opportunity to test their skills and knowledge and the chance to enhance and consolidate them. 

## 5. Discussion

The course “PERLE bed-side examination” was developed for students prior to the second state examination. This course has been held for one year now, and has been improved steadily with the help of evaluations, to better fit the students' needs. A high demand to practice the clinical examination, especially in the field of neurology and orthopaedics was shown by the inquiry. Because of this, we have developed special courses only for students right before the final state examination. These courses recapitulate practical and theoretical skills in neurology and orthopaedics. 

The strong points in favour of the course “PERLE bed-side examination” are the ratio of tutors and students (1 tutor teaches 4 students), the interactivity of the course and the flexibility to answer to every single group. In this particular setting, the special benefit of peer-teaching can be shown, which is repeatedly stated in the literature (e.g. [5]): Students rather dare to try something new and ask a lot of questions in this “sheltered” situation. In the tense situation the students are in just before the exam, this is especially valuable.

The weak points are the limitations in time that only allow an extract of the examinated topics to be conducted. Besides, it is not possible to demonstrate and practice a complete, structured clinical examination of the whole body in the setting of the course. Therefore, we are designing another course, which aims to teach a structured physical examination from head to toe. This course will be open for earlier semesters and will be the basis for “PERLE bed-side examination”.

Another weak point is the lack of tracking further development and learning enhancement of the participants. To collect data about the students' progress, a group assessment is planned. We want to see whether the students make progress in the course of the four stations.

The course was created for the students to achieve more self-assurance, so in our opinion there is no need for it to be integrated into the curriculum. But it is essential that a structured clinical examination is taught in the first clinical years. Therefore, it is important to integrate the course “clinical examination from head to toe” into the curriculum. 

Strikingly, the number of women participating decreased from 72,4% to 60,6% in the course of the following exams (spring and autumn 2015 together). The reasons for this are to be assessed, in future courses and evaluations, by asking for the students' individual reasons to visit the course.

## 6. Conclusion

With this new course concept, it was possible to meet some of the students' needs, right before their state examination. Through the evaluations we ascertained the need for a broad variety of case studies and the need for more opportunities to practice the clinical examination, especially the neurological and orthopaedic clinical examination. We will put this into action in future semesters, and we will record the students progress by further evaluation.

## Competing interests

The authors declare that they have no competing interests.

## Supplementary Material

Attachment 1

Attachment 2

Attachment 3

Attachment 4

## Figures and Tables

**Table 1:**
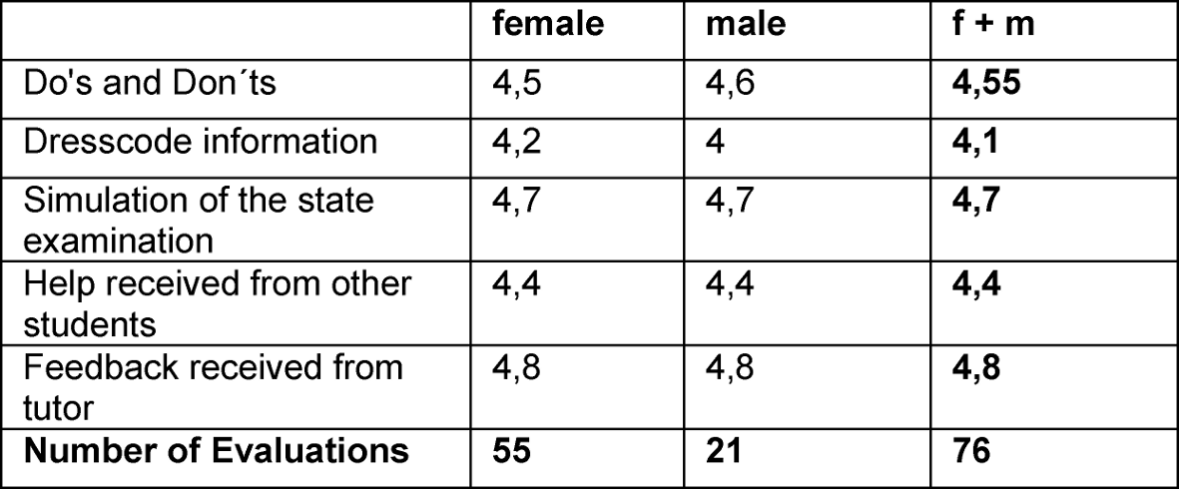
Overall evaluation; evaluation in Likert-Scale: 1=not helpful at all – 5=very helpful

**Table 2:**
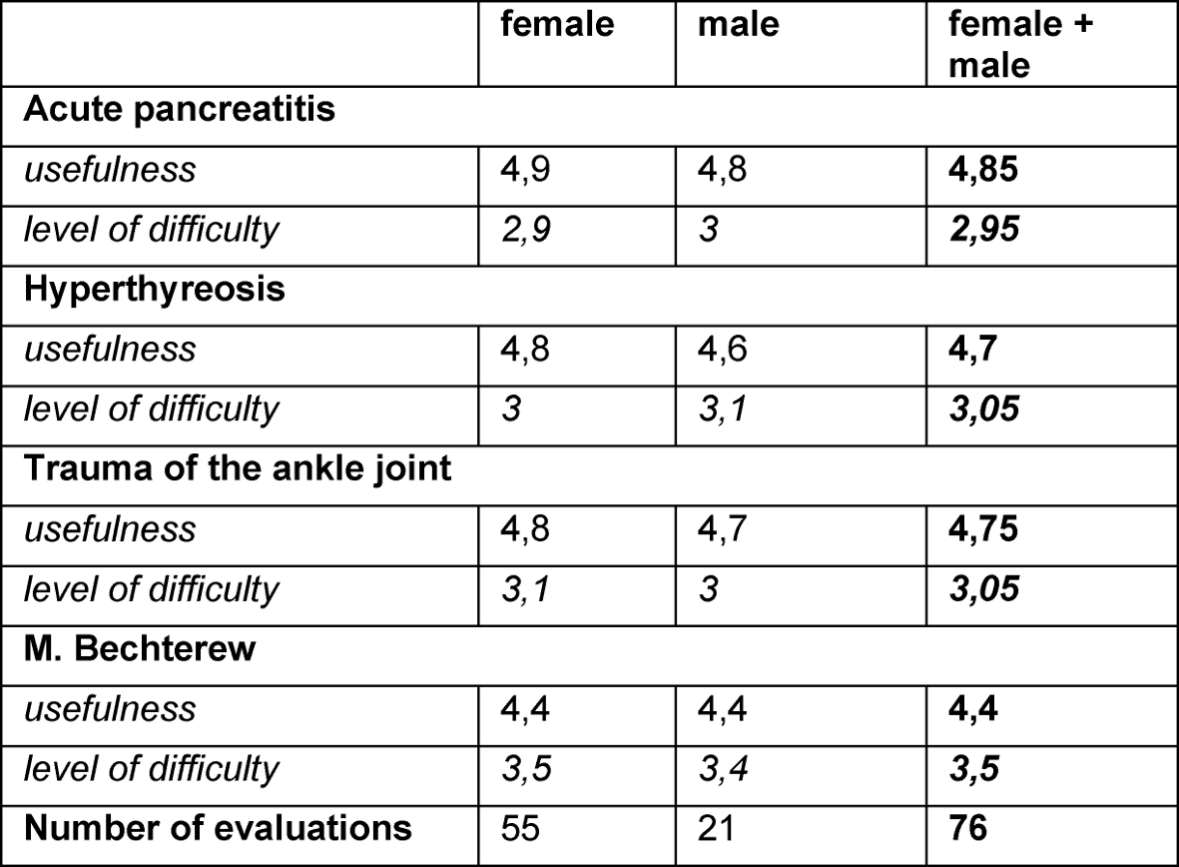
Evaluations of the case study; 1=not useful at all – very useful; 1=too easy – 5=too difficult
