# Supplementary material for: “PERLE bedside-examination-course for candidates in state examination” – Developing a training program for the third part of medical state examination (oral examination with practical skills)
Source: GMS J Med Educ. 2016 Aug 15;33(4):Doc55. doi: 10.3205/zma001054 (PMC5003133; doi:10.3205/zma001054)
Supplement: Attachment 1 [file JME-33-55-s-001.pdf]

STATION: Morbus Bechterew (Spondylitis ankylosans)

Material: examination couch, measuring tape, Edding/pen, reflex hammer

Case report:
The Tutor reads out the italic words:
A 25-year old patient visits your consultancy. He reports about pain in the back, which began gradually. Now it is so severe that he had to take a pain killer (Ibuprofen). Furthermore, he feels limited in the movement of his spine. The patient works as an administrative officer. He is of normal weight (75kg; 185cm) and works out rarely. You assume an inflammatory disease as the cause of the back pain.

Exercise 1: Please name typical symptoms that your patient with back pain, caused by an inflammatory disease, might report!

Nr.		Completely accomplished	Partially accomplished	Not accomplished
1.	Beginning before the age of 45			
2.	Gradual onset			
3.	Pain particularly in the morning with morning stiffness			
4.	Improvement during the day/with movement			
5.	Pain for more than 3 months			
6.	Radiating pain without neurological failures			
7.	Backache/buttock pain in the second half of the night			

After accomplishing the anamnesis, the patient affirms all criteria for back pain of an inflammatory cause.You assume an inflammatory spine disease, like Spondylitis ankylosans (Morbus Bechterew) or other spondyloarthropathies.

Typical for Morbus Bechterew is also a genetic predisposition (HLA-B27 positive), rheumatic factors in the blood negative, male gender, pain in the thoracolumbar transition and pain at knocking or relocation of the sacroiliac joint.

Exercise 2: Name other possible manifestation of Morbus Bechterew (extra-spinal)

Nr.		Completely accomplished	Partially accomplished	Not accomplished
1.	Peripheral arthritis (hip, knee, upper ankle joint, fingers, toes)			
2.	Uveitis anterior			
3.	Enthesiopathies (painful inflammation of the tendon insertion), like allodynia			
4.	Chest pain (Synchondritis of sternum and manubrium), pain in the pubic bone (Symphysitis)			

Exercise 3: Please examine the spine!

Nr.		Completely accomplished	Partially accomplished	Not accomplished
1.	Hygiene (hand disinfection, take off jewelry and watches)			
2.	Introduction to the patient and explaining the following steps			
3.	Inspection (torso undressed)			
4.	Testing the flexibility of the spine (forwards, sideways and rotation in the spine with the pelvis fixed)			
5.	Testing the spine for pain though percussion or compression			
6.	Assessing the lung excursion (possibly by measurment)			
7.	Assessing the flexibility of the thoracic spine (Ott sign)			
8.	Assessing the flexibility of the lumbar spine (Schober sign)			
9.	Measuring the finger-floor distance			

Tips for the examination:

- Inspection: hints for osteoporosis, hints for spondylolisthesis, erythema, swelling, hyperlordosis/hyperkyphosis etc.
- Patient bends forwards: Skoliosis? Progressive kyphosis? Possibly quantified by the distance between head and wall in a standing, maximum upright position against the wall: >0cm = pathologic
- Pain by compression: Tested by plunging from tiptoe to heel position
- Lung excursion: Measured just below the mamilla; at least 5cm; Ask the patient about pain
- Ott-sign: Measuring 30 cm distance in caudal direction from vertebra prominens (7th cervical vertebra). Mark the spot. Then the patient bends forwards to the maximum. The distance should increase at least by 2cm
- Schober-sign: Measuring 10cm distance in cranial direction from the 5th lumbar vertebra (between the two spinae iliacae post. sup.). Mark the spot. The patient bends forwards to the maximum. The distance should increase at least by 4cm.
- Finger-floor distance: around 0-10cm; negativ results are possible. Follow-up parameter.

Exercise 4: Perform an orientating neurological examination to exclude the differential diagnosis of a slipped disk!

Nr.		Completely accomplished	Partially accomplished	Not accomplished
1.	Lasègue-sign			
2.	Orientating sensory testing			
3.	Orientiating test of the muscular reflexes of the lower extremity (ATR, PTR, BTR)			
4.	Testing for Babinski-reflex			
5.	Orientating test of the muscular strength			

Tips for the examination:

- Lasègue-Sign: Sign of ischiadic stretching. The patient lies relaxed on his back with stretched legs. Flexing the stretched leg upwards causes noticable pain that radiates into the leg if Lasègue was positive.
- Sensory testing: Ask the patient about paresthisia, hypesthesia, dysesthesia. Testing the tactile sensation by simultaneously stimulating both legs by touching.
- Babinski-Reflex: Firm stimulation of the lateral plantar foot with a slightly sharp object. Positive: Dorsalextension of the big toe and spreading apart of the rest of the toes.
- Testing the muscular strenght: Standing up from the squatting position without the use of arms; Moving the tiptoe in cranial/caudal direction against resistance.

Excercise 5: Finish the physical examination by testing the Mennell-sign! Explain your approach.

Nr.		Completely accomplished	Partially accomplished	Not accomplished
1.	The patient lies on the belly			
2.	Finding the sacroiliac joint. Testing for pain by compression			
3.	Hyperextending the hip joint in dorsal direction. Doing so, fix the os sacrum with the other hand			

Tips for the examination:

- Mennell-Sign: The patient is lying on the stomach. The ipsilateral leg is held above the knee with one hand. The other hand is on the sacroiliac joint. Now do a hyperextension of the hip joint by lifting the leg in dorsal direction. If the patient shows pain in the sacroiliac joint the sign is positiv.

Deviating answers, remarks of the auditor:
