# Supplementary material for: “PERLE bedside-examination-course for candidates in state examination” – Developing a training program for the third part of medical state examination (oral examination with practical skills)
Source: GMS J Med Educ. 2016 Aug 15;33(4):Doc55. doi: 10.3205/zma001054 (PMC5003133; doi:10.3205/zma001054)
Supplement: Attachment 2 [file JME-33-55-s-002.pdf]

Dress code for the oral part of the medical state examination

	Recommended	Not recommended
**Clothing**	Males:<br>• trousers (wool, cotton, firm quality), ironed, modest colours, regular fit<br>• shirt +/- tie<br>• suit/jacket in classic style and design<br>females (possibilities):<br>• blouse or elegant top (without low neckline, shoulders and neck should be covered)<br>• blazer<br>• trouser of sporty elegance: modest colours, ironed skirt (minimum knee length; minimum length of 5cm above the knee in sitting position)<br>• dress (modest neckline and length, professional appearance)<br>• tights in modest colours<br>white coat:<br>• clean, white, no tears or holes, ironed, right fit<br>Mind your freedom of movement!	• tops that expose certain body parts by bending over (neckline, waistline)<br>• sleeveless Tops<br>• jeans/denim/leather trousers<br>• cargo pants or pants with large pockets<br>• baggy pants<br>• shorts<br>• track suits<br>• t-shirts<br>• tops with a deep neckline<br>• strapless tops<br>• hooded jackets or sweatshirts<br>• miniskirts, skirts that are too short<br>• large and/or provoking labels<br>• clothing with tears or holes<br>• clothing with too tight or too loose cut<br>• transparent tops<br>• sportswear<br>• obviously unwashed cloths
**Shoes**	• formal shoes in good condition, furbished, worn with socks<br>• closed tip, possibly with a moderate heel	• sports shoes<br>• sneakers<br>• peep toe<br>• sandals, flip-flops<br>• dirty shoes
**Body hygiene**	• well-groomed appearance, no obtrusive fragrance<br>• hair well-groomed and clean hair<br>• long hair should be tied back<br>• facial hair short and shaved<br>• possibly make up, modest in shade and style, nude look<br>• glasses without obtrusive or glaring colours<br>• Nails short and clean<br>• No nail-polish	• Obtrusive scent (fragrance, sweat, cigarette smoke)<br>• dramatic hairstyles/ hair colours<br>• hair covering the face<br>• excessive beard hair<br>• striking make-up in glaring and unnatural colours<br>• head gear (except for religious reasons)<br>• sun glasses<br>• long and unkempt fingernails<br>• nail polish, artificial or tip-elongated nails
**Jewellery**	• possibly necklace or earrings, (modest in size, appearance and colour)<br>• maximum of 1 earring per ear<br>• take off watches and rings	• large, jingling, glaring jewellery<br>• more than 1 earring per ear<br>• visible tattoos or piercings
**ID card**	• name tag should be placed visible on chest level	• no name tag<br>• distracting logos or stickers
