# Supplementary material for: “PERLE bedside-examination-course for candidates in state examination” – Developing a training program for the third part of medical state examination (oral examination with practical skills)
Source: GMS J Med Educ. 2016 Aug 15;33(4):Doc55. doi: 10.3205/zma001054 (PMC5003133; doi:10.3205/zma001054)
Supplement: Attachment 3 [file JME-33-55-s-003.pdf]

Important steps during physical examination should be performed automatically:
put the bed in working height - always examine the patient from the **RIGHT SIDE** – explain your procedures to the patient – introduce yourself to the auditors – introduce the auditors to the patient

Usually, transcripts of former exams provide a sufficient preparation. Especially the 4th subject is more likely to be assessed in a rather basic level or certain topics are even specified for preparation.

Don´t say everything you know right at the beginning. Hold some of your knowledge back for further inquiries.
Only name issues you can explain afterwards in specific inquiries.

„Usually, you won`t fail the examination. Even if you said something wrong, you can still obtain an excellent mark "

"The auditors usually try to maintain a pleasant atmosphere and they don`t want to expose you to ridicule"

DON`T

- o Appearing to nervous and anxious
- o Smell of cigarette smoke
- o Protrude obviously from the rest of your group
- o Speak in maybes and subjunctives
- o Use a questioning intonation in your sentences

- ✓ Appear open minded and friendly
- ✓ Be on time
- ✓ Comply with the formalities: **NO EXAM WITHOUT ID-CARD AND EXAM PAPERS**
- ✓ Keep eye contact with the auditor
- ✓ Keep a natural, open position. Your hands should not be in your pockets/face/mouth/hair
- ✓ Shake hands with every auditor
- ✓ Introduce yourself to the auditors before the exam
  Make an appointment as a group with your auditor (if he is willing to do appointments)
- ✓ Act like you are in an expert discussion; act like a capable interlocutor, not like student and teacher
- ✓ Describe everything you see during inspection – "think out loud"
- ✓ Use the definite form "I will examine…now" instead of the subjunctive "I would like to examine.."
- ✓ Specific questions require a specific answer
- ✓ If you didn`t understand the question, ask for a different verbalisation
- ✓ Present your knowledge in a knowing, clear and calm voice
- ✓ Go from general to specific. Especially basic knowledge and procedures (anamnesis, physical examination, basic tests) are important. You should know emergency situations and know how to proceed adequately.
- ✓ **ACT CONFIDENTLY EVEN IN CLUELESSNESS!**
- ✓ Coordinate breaks, such as going to the toilet, with the auditors. Don`t just leave the room
- ✓ Check the patient history/record/preliminary findings carefully
- ✓ Mark the filled anamnesis form with your name

How to act if you have no clue?
NEVER say "I have no idea"
Rather try:
"I didn´t concentrate very intensely on this subject any more "

"Regrettably, this was not a subject I put emphasis on in the hospital. Could you maybe give me some key words to prompt my memory?"

BUT if you definitely don`t know anything about it, don`t hum and haw.
A good option would be: "I´m sorry, I don´t get the idea right now"
But you have to consider, that the next question should better be answered right away. Otherwise you can stall for some time while the auditors try to help you find the right answer.

„It´s the overall impression, that counts in the end. With a self-confident manner you can make up for not knowing something from time to time."

DON´T FORGET:
Ironed white coat
Examination tools (lamp, stethoscope, reflex hammer etc.)
ID-CARD
EXAM PAPERS
