# Supplementary material for: “PERLE bedside-examination-course for candidates in state examination” – Developing a training program for the third part of medical state examination (oral examination with practical skills)
Source: GMS J Med Educ. 2016 Aug 15;33(4):Doc55. doi: 10.3205/zma001054 (PMC5003133; doi:10.3205/zma001054)
Supplement: Attachment 4 [file JME-33-55-s-004.pdf]

Evaluation:

"PERLE bed-side examination"
Participants: Students in their final year

☐ male ☐ female ____ Semester ________________ Date

Thank you very much for participating in the course "PERLE bed-side examination"! To improve the course and to meet the needs of students in the final year, we would like you to answer the following questions:

The „Do's and Don'ts" were:

	--	-	0	+	++	
not helpful at all	1	2	3	4	5	very helpful

Information about the dress code was:

not helpful at all	1	2	3	4	5	very helpful

The simulation of the state examination was:

not helpful at all	1	2	3	4	5	very helpful

The case study „acute pancreatitis" was:

not useful at all	1	2	3	4	5	very useful
too easy	1	2	3	4	5	too difficult

The case study „ hyperthyreosis" was:

not useful at all	1	2	3	4	5	very useful
too easy	1	2	3	4	5	too difficult

The case study „trauma of the ankle joint" was:

not useful at all	1	2	3	4	5	very useful
too easy	1	2	3	4	5	too difficult

The case study „M. Bechterew" was:

not useful at all	1	2	3	4	5	very useful
too easy	1	2	3	4	5	too difficult

The help received from other students was:

not helpful at all	1	2	3	4	5	very helpful

The feedback from the tutor was:

not helpful at all	1	2	3	4	5	very helpful

Do you have any wishes, ideas or criticism regarding the course?
